# GI-REASONS: a novel 6-month, prospective, randomized, open-label, blinded end point (PROBE) trial

**DOI:** 10.1186/ar3664

**Published:** 2012-02-09

**Authors:** Byron Cryer, Chunming Li, Lee S Simon, Gurkirpal Singh, Martin J Stillman, Manuela Berger

**Affiliations:** 1 University of Texas Southwestern Medical Center, Dallas, TX, USA; 2 Pfizer Inc. New York, NY, USA; 3 SDG, LLC, Cambridge, MA, USA; 4 Stanford University, Palo Alto, CA, USA; 5 Hennepin County Medical Center, Minneapolis, MN, USA

## Background

The GI Randomized Event and Safety Open-Label NSAID Study (GI-REASONS) was a novel prospective, randomized, open-label, blinded end point (PROBE) study that measured adjudicated clinical outcomes throughout the GI tract. It was designed to assess if celecoxib use in patients with osteoarthritis (OA) at moderate GI risk (≥55 y) is associated with a lower incidence of clinically significant upper and lower GI events compared to nsNSAIDs, with/without proton-pump inhibitors (PPIs), in standard US clinical practice.

## Materials and methods

8067 OA patients were randomized 1:1 for 6-mos with celecoxib or a nonselective (ns)NSAID, stratified by *H pylori *status. The primary end point was a composite of adjudicated clinically significant upper and lower GI events. Aspirin use was not permitted. Treatment doses could be adjusted per US prescribing information. Patients randomized to the nsNSAID arm could switch between nsNSAIDs; however, crossover between treatment arms was not allowed. PPIs and histamine-2 receptor antagonists (H_2_RAs) were prescribed at the providers' discretion.

## Results

4035 celecoxib and 4032 nsNSAID patients were randomized and included in the ITT analyses. Baseline demographics were similar. Overall, significantly more nsNSAID users met the primary end point at 6 mos (OR, 1.82; 95% CI 1.31-2.55; *p *= 0.0003; Table 1). The most commonly used nsNSAIDs were meloxicam (42%), naproxen (21%), diclofenac (20%) and nabumetone (14%). 2596 celecoxib (64.3%) and 2611 (64.8%) nsNSAID users completed the study. 189 patients were lost to follow-up (LTFU; 2.1% celecoxib and 2.6% nsNSAID). Attributing the primary end point to all LTFU patients (worst-case sensitivity analysis), celecoxib remained superior (OR 1.46; 95% CI 1.18-1.82; *p *= 0.0006). AEs, SAEs and discontinuations were similar in both treatment groups. 23% of celecoxib and 24% of nsNSAID patients used a PPI (*p *= NS). Moderate to severe abdominal symptoms were experienced by 94 (2.3%) celecoxib and 138 (3.4%) nsNSAID patients (*P*<.01).

**Table 1:** Clinically significant upper and lower GI events: primary analysis

		Celecoxib		nsNSAID	
	
		N	Patients With Event n (%)	N	Patients With Event n (%)
**All patients**		4035	54 (1.3)	4032	98 (2.4)

***H pylori *status**	**Positive**	1401	25 (1.8)	1386	34 (2.5)
	
	**Negative**	2634	29 (1.1)	2646	64 (2.4)

**OR (95% CI); P value**		1.82 (1.31-2.55); *p *= 0.0003			

## Conclusion

Celecoxib use had a lower risk of clinically significant upper and lower GI events than nsNSAIDs. A major strength of this study is its PROBE design. Simple inclusion and exclusion criteria allowed for a broad patient population of moderate GI risk. Switching among nsNSAIDs and allowing for dose adjustments, along with use of PPIs and H_2_RAs as needed, more closely reflects daily clinical practice. GI-REASONS demonstrates the improved GI safety profile of celecoxib throughout the GI tract in patients treated in a "real-world" setting.

